# Mitochondrion-toxic drugs given to patients with mitochondrial psychoses

**DOI:** 10.1186/1744-9081-8-45

**Published:** 2012-08-29

**Authors:** Josef Finsterer

**Affiliations:** 1 KAR, Postfach 20, Vienna, 1180, Austria

## Commentary

**Figure 1:**
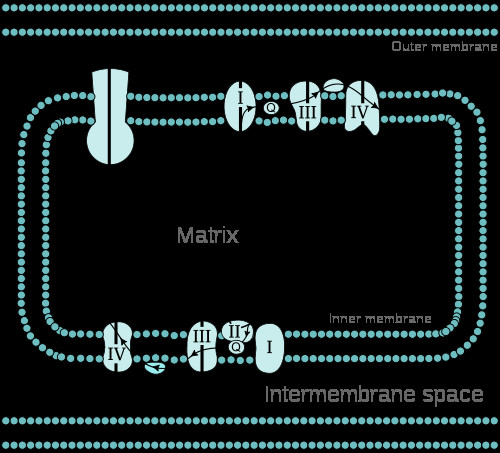
**Eukaryotic respiratory chain located at the inner mitochondrial membrane.** While electrons are transported horizontally along all respiratory complexes, protons are vertically pumped into the inter-membrane space by complexes I, III, and IV. Via complex V protons are pumped into the matrix to react with the electrons to ATP and H2O (from Wikipedia)

The cause and pathophysiology of mood and psychotic disorders, including unipolar depression, bipolar disorder, and schizophrenia, is variable. Several scenarios have been proposed to explain the development of psychoses, one of which is mitochondrial dysfunction (mitochondrial mood and psychotic disorders) [1,2]. Impaired mitochondrial pathways, which may be associated with mood and psychotic disorders, include the respiratory chain (Figure 1) [1,3], the pyruvate-dehydrogenase complex, the 2-ketoglutarate dehydrogenase [4], and the polyol pathway [5]. Disturbance of the respiratory chain is the most frequent cause of mitochondrial mood and psychotic disorders. Mood and psychotic disorders in patients with mitochondrial disorders may be the sole manifestation, or one among other manifestations, of a mitochondrial disorder. Mood and psychotic disorders may be the dominant feature of the phenotype or a collateral finding, and may occur in syndromic as well as non-syndromic [5,6] mitochondrial disorders. 

A recent study, published in this journal [1], aimed to assess psychiatric abnormalities in mitochondrial disorders with a proven mtDNA defect [1]. Twelve patients had a syndromic mitochondrial disorder (mitochondrial encephalomyopathy, lactic acidosis, and stroke-like episodes (MELAS), myoclonic epilepsy with ragged red fibers (MERRF), neuropathy, ataxia, and retinitis pigmentosa (NARP), progressive external ophthalmoplegia (PEO), Kearns-Sayre syndrome (KSS)) and seven patients had a non-syndromic mitochondrial disorder. The frequency of psychiatric diagnoses among these patients was reported to be 47% [1]. Psychiatric conditions may be even more common in other studies and include major depression, agoraphobia and/or panic disorder, generalized anxiety disorder, social anxiety disorder, or psychotic syndromes [7]. The medication these patients took at inclusion was provided for only nine patients. Among these drugs, however, several are reported to be mitochondrion-toxic. Two patients were medicated with valproic acid, two with quetiapine, and one each with carbamazepine, atorvastatin, mirtazepine, metformin, and trazodone [1].

The study did not address whether, and to what degree, anti-psychotic or other medication may worsen the underlying defect due to mitochondrion-toxicity of the applied medication [1]. This is important since deterioration of the clinical presentation may not only be due to worsening of the underlying metabolic defect, but also due to mitochondrion-toxicity of the applied anti-psychotic medication. Since a number of mitochondrion-toxic drugs are used to treat mood and psychotic disorders and may worsen the underlying metabolic defect, it is essential to exclude a mitochondrial metabolic defect before applying these agents. In addition to anti-psychotic drugs, patients with mitochondrial disorders and mood and psychotic disorders may also take other drugs, which may be mitochondrion-toxic. For a number of anti-psychotic and other drugs, however, it is not well known whether they are truly mitochondrion-toxic, neutral or, rather, mitochondrion-protective.

Some agents described in the study have been reported to cause severe, sometimes even fatal, adverse reactions, such as valproic acid, which may cause irreversible liver failure, particularly in patients carrying POLG1 mutations [8]. Other drugs may be mitochondrion-toxic without obvious major clinical side effects, such as atorvastatin, which reduces the coenzyme-Q content and generally decreases mitochondrial functions [9], mirtazepine, which decreases complex-I activity [10], metformin, which inhibits complex-I of the respiratory chain [11], quetiapine, which inhibits complex-I [12], or trazodone, which collapses the mitochondrial membrane potential and imposes oxidative stress [13]. Antipsychotic medication may not only affect the function of respiratory chain complexes, but also activity of the pyruvate-dehydrogenase complex [14]. Some of the drugs applied may also have a protective effect on mitochondrial functions, such as vinpocetine [15], trimetazidine [16], sertraline [17], levetiracetam [18], bisoprolol [19], or enalapril [20].

Based on these considerations, it is advisable that patients with mitochondrial disorders receive special attention when treated with agents whose effects on mitochondria are uncertain or definitively toxic [21]. Not only may anti-psychotic compounds be mitochondrion-toxic but so could be a number of other agents used in the daily routine. Since mitochondrial disorders are gaining increasing attention and thus being diagnosed more often, care has to be taken when selecting drugs for these patients. Since some patients may be highly sensitive to various compounds, these patients should be treated like patients with myasthenia gravis, who also react to contra-indicated medication with severe, occasionally fatal side-effects. Animal and human studies on the compatibility of agents with already disturbed mitochondrial metabolism, however, are required to find out which of the drugs are tolerated by patients with mitochondrial disorders and which are toxic to them.
